# A life‐threatening presentation of postgastrectomy exocrine pancreatic insufficiency: A case report

**DOI:** 10.1002/ccr3.8037

**Published:** 2023-10-10

**Authors:** Shiva Rahimipour Anaraki, Milad Gholizadeh Mesgarha, Leyla Bahadorizadeh, Morteza Hassanzadeh

**Affiliations:** ^1^ Faculty of Medicine Iran University of Medical Sciences (IUMS) Tehran Iran; ^2^ Antimicrobial Resistance Research Center, Institute of Immunology and Infectious Diseases Iran University of Medical Sciences (IUMS) Tehran Iran; ^3^ School of Medicine, Department of Internal Medicine Colorectal Research Center, Rasoul‐E‐Akram Hospital, Iran University of Medical Sciences Tehran Iran

**Keywords:** anasarca, gastric cancer, gastrointestinal surgery, hypoalbuminemia, pancreatic insufficiency, steatorrhea

## Abstract

**Key Clinical Message:**

Physicians must be alert for the exocrine pancreatic insufficiency diagnosis through the follow‐up of postgastrectomy patients, regardless the severity and lag time. Urgent albumin and pancreatic enzyme replacement should be considered when diagnosed.

**Abstract:**

It is documented that exocrine pancreatic insufficiency (EPI) can develop after gastrectomy. Steatorrhea, malnutrition, and weight loss are common symptoms of the disease; however, it is usually mild to moderate postgastrectomy. This article reports a case of EPI manifested by hypoalbuminemia leading to dyspnea and anasarca, which are not typical symptoms of postgastrectomy EPI. A 61‐year‐old man with a history of gastric adenocarcinoma treated by total gastrectomy and chemoradiotherapy was admitted to the hospital with dyspnea and anasarca. Despite being diagnosed as a case of malignancy recurrence in another hospital, based on the symptoms described, no evidence of malignancy was found. His ascites and pleural effusion were determined to be caused by hypoalbuminemia. In addition, he claimed steatorrhea, and his stool elastase was lower than expected. EPI was diagnosed based on his medical history, paraclinical tests, and examinations. He remained asymptomatic for 1 year after being treated with albumin and pancreatic enzymes. Postgastrectomy EPI may be severe enough to cause steatorrhea or hypoalbuminemia. Hence, regardless of the severity of the presentation, physicians must be alert for this diagnosis throughout the follow‐up of patients with a history of gastrectomy. Urgent albumin and pancreatic enzyme replacement should be considered when diagnosed.

## Graphical abstract

Severe exocrine pancreatic insufficiency has the potential to be fatal; therefore, its diagnosis requires vigilance!

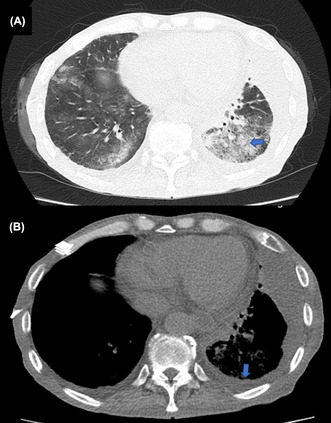

## 1 BACKGROUND

Gastric cancer is the third most prevalent cause of cancer‐related death worldwide and the fifth most common cancer overall.^1^ Survival rates for gastric cancer are improving, particularly since the introduction of neoadjuvant therapy; however, surgery remains the mainstay of curative treatment; total gastrectomy is the acknowledged surgical treatment for gastric cancer.^2^, ^3^ Up to 90% of patients who have undergone gastrectomy report experiencing gastrointestinal symptoms, including dysphagia, abdominal distension, pain, diarrhea, and, vomiting leading to malnutrition, which is associated with a poor prognosis.^4^, ^5^, ^6^

One of the causes of maldigestion is exocrine pancreatic insufficiency (EPI), a disorder characterized by insufficient or abnormal production or activity of pancreatic juice. EPI can lead to steatorrhea, stomach distension, flatulence, and weight loss.^7^ As it is well established that EPI caused by gastrectomy with duodenal continuity is often mild to moderate and not severe enough to cause steatorrhea, diagnosing patients with atypical presentation can be difficult and even result in misdiagnosis.^8^ In light of this, we discuss a patient with postgastrectomy EPI who presents with anasarca, pleural effusion, and dyspnea due to postgastrectomy EPI.

## 2 CASE PRESENTATION

A 61‐year‐old man with a history of gastric adenocarcinoma presented to the hospital with a history of fever, anasarca, and exacerbation of dyspnea. He was treated by total gastrectomy, six sections of chemotherapy with capecitabine and oxaliplatin regimen, and 30 sections of radiotherapy, 2 years prior. Despite not having his medical record, he mentioned that his gastric cancer had been followed up every 3 months using computed tomography and laboratory samples. Additionally, he cited a high‐protein, low‐carbohydrate diet according to the nutritionist's recommendation. In the follow‐up process, he presented with progressive dyspnea. He was admitted to another hospital 4 months ago, and according to the left pleural effusion, a pigtail catheter was inserted, and he was discharged with the diagnosis of cancer recurrence and palliative therapy. He was under the treatment of albumin, furosemide, meropenem, and levofloxacin from a month until 2 days ago, when he was administered Intralipid and amino acid, which exacerbated his symptoms. He was brought to hospital with fever, functional class 3 dyspnea, and edema. At the presentation, he exhibited a fever of 38, a respiratory rate of 25, a pulse rate of 110, and a blood pressure of 100/70. Lung auscultation revealed diminished lung sounds, particularly in the right lung. There was no evidence of jugular vein distension, and heart sounds were normal. During the examination of the abdomen, the abdomen was distended. Shifting dullness was positive, however; no tenderness was detected. Additionally, he had edema in his legs and scrotum.

**FIGURE 1:**
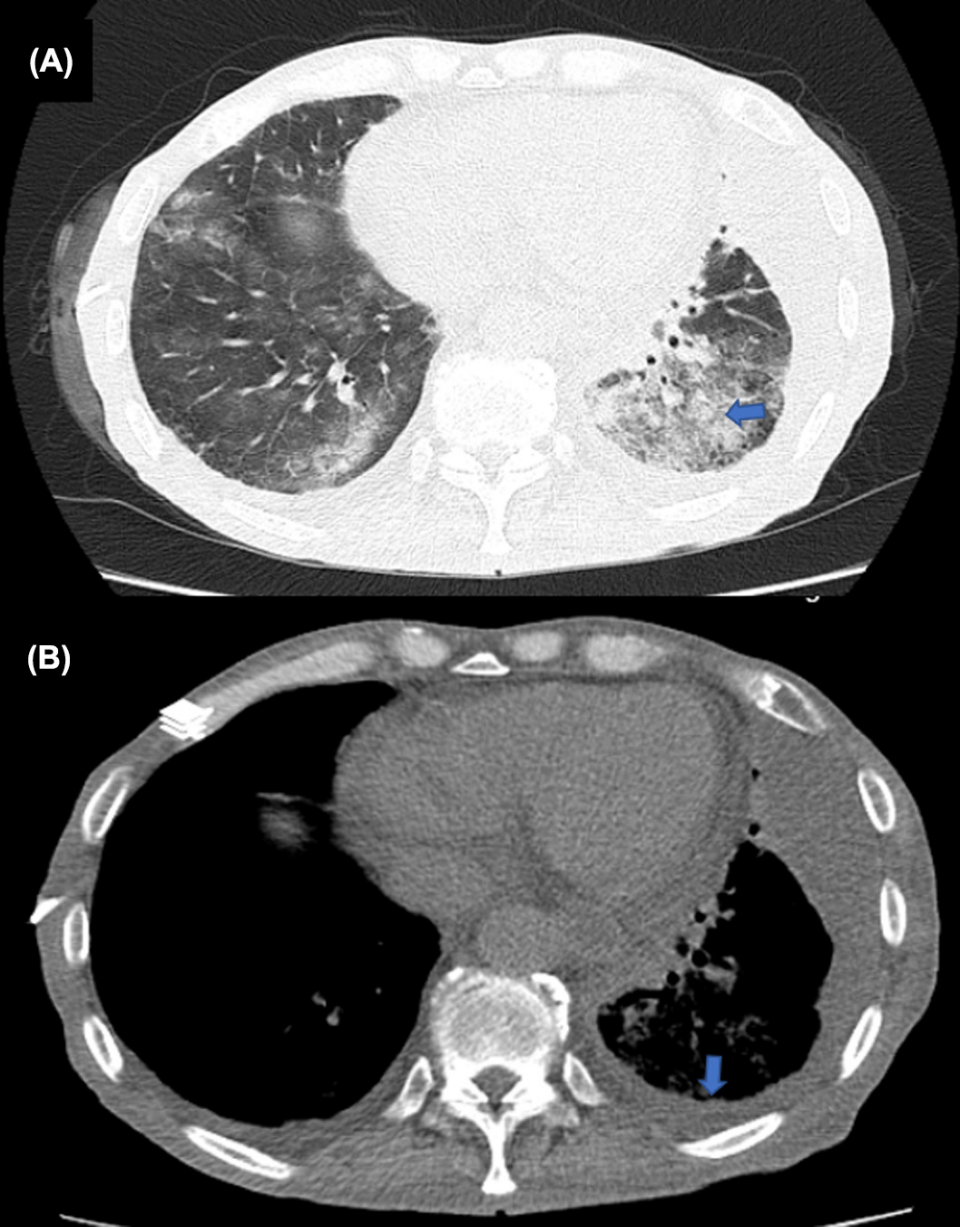
It depicts chest CT without contrast, (A) demonstrates lung window, sub pleural patchy nodular grand glass opacities of left lower lobe is seen, which is consistent with atypical pneumonia (rule out metastasis); (B) demonstrates mediastinal window, mild pleural effusion is seen.

On admission, his serum albumin was 2.2 g/dL, his white blood cell count was 28.8, his hemoglobin was 6.8 (MCV:95), and his platelet count was 101. All other laboratory results, including liver, renal, and thyroid function tests, amylase, lipase, LDH, electrolytes, and 24‐h urine test, were within normal range except for a 3+ CRP. His spiral chest tomography revealed mild left‐sided pleural effusion, segment collapse of the left lower lobe, and patchy nodular grand glass opacities of the left lower lobe (Figure 1). Tomography of the abdomen and pelvis revealed ascites and mucosal thickening of jejunal loops. As a result, abdominal paracentesis and thoracocentesis were carried out. The thoracocentesis demonstrated transudate effusion. The ascetic fluid analysis revealed a high SAAG and low protein analysis, with no PMN and negative culture test.

Consequently, antibiotic treatment was commenced, and left chest pigtail catheterization and peritoneal catheterization were performed on the patient, which caused his dyspnea to improve, and the leukocytosis and patchy nodular grand glass opacities to disappear. Due to intestinal loop thickening, the patient underwent magnetic resonance enterography, which revealed no lymphadenopathy or abdominal mass. In addition, he underwent bronchoalveolar lavage and lung biopsy to evaluate lung metastases, which were negative. Upon admission, his laboratories revealed pancytopenia. As a result, serum electrophoresis, bone marrow aspiration, and biopsy were performed per hematology group consultation, although none of them demonstrated abnormal findings. Cobalamin and folate were prescribed for him with a suspicion of megaloblastic anemia due to gastrectomy, and patient blood cells improved.

Finally, due to the unknown cause of hypoalbuminemia, a more detailed medical history was obtained, during which he stated a history of steatorrhea and flatulence. The elastase level in his stool was recorded as 44 (normal range >200). Anti‐TTG, anti‐gliadin IgG, and alpha‐one antitrypsin were all negative, and neither the patient's medical history nor imaging results were consistent with a low stool elastase level. With suspicion of EPI, 300 mg of pancrelipase three times daily was administered orally. The patient's symptoms and laboratory values returned to near‐normal levels, and he remained asymptomatic under treatment for 1 year.

## 3 DISCUSSION

EPI following complete gastrectomy is a known complication. The significance of this complication has become more evident recently in the context of growing trends in bariatric surgery, as individuals undergoing these procedures are at a higher risk of developing postoperative EPI.^9^, ^10^ The pathophysiological origins of this complication are not entirely understood; however, multiple factors contribute to the pathogenesis of EPI after gastrectomy. The pancreatic denervation due to lymph node resection, the reduction of pancreatic neural stimulation due to the loss of antro‐fundic and duodeno‐fundic reflexes, food's rapid transit into the small intestine due to loss of gastric reservoir, and abnormalities in the release of hormones like cholecystokinin are hypothesized as likely causes.^4^, ^10^, ^11^ The severity of EPI due to gastrectomy with duodenal continuity is typically mild to moderate and not severe enough to cause steatorrhea, which demands a 90% reduction in lipase^8^; however, our patient presented with steatorrhea and a life‐threatening clinical condition due to EPI following gastrectomy.

The patient's pleural and abdominal fluids were inconsistent with malignancy or cancer recurrence. There were no indications of the causes of transudate pleural effusion, such as heart failure, cirrhosis, or nephrotic syndrome, nor of the causes of high SAAG low protein ascites, such as portal hypertension or cirrhosis; thus, hypoalbuminemia remained as the underlying cause. All other potential underlying causes of hypoalbuminemia were ruled out except for steatorrhea. Steatorrhea accompanied by low stool elastase and normal imaging in the absence of coeliac disease, diabetes, inflammatory bowel disease, and small intestine overgrowth increased the likelihood of a EPI diagnosis. Finally, the fact that the patient remains asymptomatic while taking pancreatin lends credence to EPI as the underlying cause.

The lone case of hypoalbuminemia, steatorrhea, and anasarca due to EPI after total gastrectomy for cancer was reported in 2019, which occurred 18 years after gastrectomy.^12^ This is the second case with a severe presentation due to EPI, only 2 years after total gastrectomy for cancer. Due to the rarity of severe postgastrectomy EPI, suspecting this uncommon clinical setting is challenging, resulting in diagnostic delay or misdiagnosis. In the case of our patient, there was no evidence of cancer recurrence; however, in the absence of a recognized explanation for the patient's clinical presentation, a misdiagnosis occurred, which could have led to patient's mortality. Nevertheless, an accessible treatment rendered him stable for 1 year. Similarly, diagnosing EPI is challenging in the context of bariatric surgery.^10^ The increasing prevalence of bariatric surgery and the limited number of documented patients with severe EPI after bariatric surgery highlight the significance of this complication even more.^13^ Hence, it is crucial to consider severe EPI in all patients undergoing gastrectomy, although mild or moderate EPI is more prevalent. Furthermore, EPI can occur over a wide range of time following the gastrectomy, from a month to 18 years. Hence, postgastrectomy EPI must be considered a probable diagnosis in patients with suspicious symptoms and a history of gastrectomy, regardless of the time of gastrectomy.^12^, ^14^

Pancreatic supplementation enhanced nutritional status and quality of life postgastrectomy in patients with a history of gastric cancer, according to small clinical trials.^15^, ^16^ Other researchers, however, considered the benefits of this treatment to be minor and unnecessary following gastric resection surgery.^4^, ^11^ In light of these two cases of severe EPI following gastrectomy, clinicians must be vigilant for deteriorating EPI symptoms on long‐term follow‐up, which may necessitate adequate investigations followed by pancreatic enzyme replacement therapy.

## 4 CONCLUSION

Postgastrectomy EPI may be severe enough to cause steatorrhea, hypoalbuminemia, ascites, and pleural effusion in patients with gastric cancer who have undergone total gastrectomy. Consequently, regardless of the severity and lag time of the initial presentation, clinicians must be alert for a diagnosis of EPI throughout long‐term follow‐up of patients with a history of gastrectomy. Urgent albumin and pancreatic enzyme replacement should be considered when diagnosed.

## AUTHOR CONTRIBUTIONS

**Shiva Rahimipour Anaraki:** Writing – original draft. **Milad Gholizadeh Mesgarha:** Writing – original draft. **Leyla Bahadorizadeh:** Conceptualization; supervision. **Morteza Hassanzadeh:** Supervision.

## CONFLICT OF INTEREST STATEMENT

The authors declare that they have no conflict of interest.

## CONSENT

Written informed consent was obtained from the patient to publish this report in accordance with the journal's patient consent policy.

## Data Availability

The data that support the findings of this study are available from the corresponding author, upon reasonable request.
